# Enactive and simondonian reflections on mental disorders

**DOI:** 10.3389/fpsyg.2022.938105

**Published:** 2022-08-03

**Authors:** Enara García, Iñigo R. Arandia

**Affiliations:** IAS Research group, Department of Philosophy, University of the Basque Country, San Sebastián, Spain

**Keywords:** enactive cognition, mental disorders, normativity, Simondon, embodiment, metastability, sense-making, adaptivity

## Abstract

As an alternative to linear and unidimensional perspectives focused mainly on either organic or psychological processes, the enactive approach to life and mind—a branch of 4-E (embodied, embedded, enactive, extended) cognitive theories—offers an integrative framework to study mental disorders that encompasses and articulates organic, sensorimotor, and intersubjective dimensions of embodiment. These three domains are deeply entangled in a non-trivial manner. A question remains on how this systemic and multi-dimensional approach may be applied to our understanding of mental disorders and symptomatic behavior. Drawing on Gilbert Simondon’s philosophy of individuation (focusing particularly on the concepts of *tension*, *metastability*, and *preindividual*), we provide some enactive conceptual tools to better understand the dynamic, interactive, and multi-dimensional nature of human bodies in mental disorders and psychopathological symptoms. One of such tools cursiva is sense-making, a key notion that captures the relational process of generating meaning by interacting with the sociomaterial environment. The article analyzes five aspects related to sense-making: temporality, adaptivity, the multiplicity of normativities it involves, the fundamental role of tension, and its participatory character. On this basis, we draw certain implications for our understanding of mental disorders and diverse symptoms, and suggest their interpretation in terms of difficulties to transform tensions and perform individuation processes, which result in a reduction of the field of potentialities for self-individuation and sense-making.

## Introduction

The biopsychosocial model proposed by Engel (1977) offered relevant ideas to go beyond reductionist approaches in medicine and psychopathology. However, after decades of research and clinical practice following and developing this model, important limitations have been made explicit in diverse fields, including psychiatry: the tendency to prioritize the biological domain in clinical practice, the fragmented application of the “bio,” the “psycho” and the “social,” the downplay of subjective experiences, and the lack of strong theoretical foundations to continue developing the model, among other limitations (Ghaemi, 2011; Stilwell and Harman, 2019; Mescouto et al., 2022; Aftab and Nielsen, 2021). The enactive approach to life and mind has been proposed as an alternative to overcome these limitations (Stilwell and Harman, 2019; de Haan, 2020). The notion of embodiment, based on the operational concept of *autonomy*, allows us to investigate human bodies as a complex set of intertwined processes at organic/biological, sensorimotor/psychological, and intersubjective/social dimensions. This framework offers a variety of other operational concepts (e.g., sense-making, agency or adaptivity) that are relevant in health research and practice. At the same time, the enactive approach is grounded in phenomenology, dynamical systems and organizational approaches to biology, offering a solid theoretical background to further develop concepts and ideas and deepen the investigation of human experience (Thompson, 2010; Di Paolo et al., 2017, 2018; Fuchs, 2017; Gallagher, 2017; Varela et al., 2017). Moreover, the enactive approach overcomes several theoretical assumptions that are often implicit in research and clinical practice which often represent a source of additional limitations. The continuity between life and mind and the deep entanglement among dimensions of embodiment overcomes pervading dualisms (i.e., the strict separation of mind and body, physiology and psychology). Individuals are considered as embodied agents rather than as passive bodies that simply react to external stimuli in a predictable and deterministic manner. In contrast with the widespread methodological individualism—the tendency to investigate isolated individuals where the environment plays a secondary modulatory role—human bodies are considered to be constitutively social^1^. Another key aspect is that subjective experience is not relegated in favor of objective measures. Instead, the goal is to seek complementarities and “mutual enlightenment” between first-person and third-person approaches (Gallagher, 1997).

Enactive ideas have been applied in the study of a variety of mental disorders, such as Obsessive-Compulsive Disorder (OCD, de Haan et al., 2013), autism (De Jaegher, 2013) or schizophrenia (Kyselo, 2016; Fuchs and Röhricht, 2017). Other works have developed and applied a systematic enactive model of mental disorders (e.g., Colombetti, 2013; de Haan, 2020; Maiese, 2022; Nielsen, 2020). The path toward an enactive articulation of mental disorders is open and fruitful, but far from being complete. In this work, we provide some reflections on certain topics in mental disorders that we think deserve more attention and clarification, namely temporality, relational aspects (including adaptive and participatory processes), and the integration of multiple normativities through tension.

In this work, we will complement existing enactive proposals on mental disorders by including some concepts from the philosophy of individuation developed by Simondon (1958/2020). The reason to introduce Simondon’s philosophy within the debate is twofold. First, he put forward a comprehensive processual and relational ontology—alternative to Whitehead’s metaphysics—that is highly compatible with enactive principles [as already suggested in James (2020), Dereclenne (2021), and Di Paolo (2021)]. Second, he provided a theoretical framework, including concepts such as *individuation, metastability*, *preindividual*, or *transindividual* that serves to interpret mental disorders in terms of processes occurring at multiple dimensions and timescales^2^ and can be inspiring to complement the current enactive theory. Simondon describes living organisms as unfinished entities in an active process of individuation—which we call self-individuation in analogy with the enactive notions of self-production and self-distinction. Self-individuation, however, does not entail a separate, already constituted self externally driving the individuation process, but it underscores the dynamic and historical integration of a variety of conflicting norms that human bodies must continuously manage. Tensions emerge from multiple regulatory demands that pull the system simultaneously in diverse directions, resulting in some degree of incompatibility or conflict between diverging tendencies. This description resonates with the enactive conception of living organisms as tensioned beings struggling between self-maintenance and self-differentiation. This basic tension is a fundamental aspect of living organisms, and not unique to pathological situations. As we will argue, understanding the living being as holding inherent generative tensions fosters a change in our perspective on the meaning of symptoms. Symptoms are no longer understood as deviations from an optimal state, or difficulties in recovering a homeostatic stationary state, but as mechanisms to maintain the tensioned integrity of the individual and as demands for further changes and individuations. We suggest that symptoms and disorders are related to excessive tension and problems or blockages to transform them. Far from providing an exhaustive and definitive enactive definition of mental disorders and symptoms, our goal is to share some preliminary reflections and to provide examples to contribute to existing work, clarifying potential misunderstandings and emphasizing the processual and relational character of mental disorders. Possible implications for clinical practice are briefly discussed.

We begin by introducing the enactive framework and the main concepts that will be employed in this work. We complement these enactive ideas by briefly presenting the philosophy of individuation as developed by Gilbert Simondon and a few notions from his processual ontology. Then, we introduce mental disorders as disorders of sense-making, and we take on our reflections based on five desiderata. (1) Sense-making is a process, which necessarily includes dynamic and historical aspects. (2) Sense-making is relational, and it is better understood in terms of adaptivity rather than as adaptation. (3) Sense-making implies a dynamic integration of multiple norms. (4) Sense-making is a form of individuation, which operates through relatively abrupt transformations of tensions. (5) Sense-making is participatory, which shows the necessity of looking at social interactions and relational patterns as playing a constitutive role in certain symptoms and disorders.

## The enactive approach to life and mind

The enactive framework is a branch of the 4E cognition theories (embodied, embedded, extended, and enactive; Newen et al., 2018) that advocates for a naturalistic approach to life and mind. It is fundamentally influenced by organizational approaches to biology, phenomenology, pragmatism, and dynamical systems theory (Thompson, 2010; Di Paolo et al., 2017, 2018; Fuchs, 2017; Gallagher, 2017; Varela et al., 2017). The application of enactive ideas to psychopathology and psychotherapy has a long-standing tradition (Colombetti, 2013; Röhricht et al., 2014; Gallagher and Payne, 2015; Gallagher, 2018; de Haan, 2020; Maiese, 2022), and it is object of discussion in current academic debates (e.g., de Haan, 2021; Nielsen, 2021; García, 2022). Cognition, from an enactive perspective, is defined as s*ense-making*, which refers to the possibility of the living organism to meaningfully interact with the environment—which in the case of humans, is a socio-material environment—according to its own needs and goals. The enactive concept of sense-making is coextensive to life^3^ and encompasses all mental phenomena, including the affective, experiential, sensorimotor (behavioral), cognitive, and intersubjective domains. Sense-making, from this approach, is always relational and depends on the co-determination or coupling with the environment. Accordingly, mental disorders have been proposed to be disorders of sense-making (de Haan, 2020). In this way, the enactive conception of mental disorders moves away from internalist and individualistic perspectives and attempts to integrate affective, interactive, social, existential, systemic, and environmental aspects in its definition. One of the fundamental aspects of sense-making is that it is a normative process which is co-extensive with the self-individuation of the living being. It is thus grounded in its biological organization.

Proponents of the enactive approach describe living organisms as autonomous (autopoietic) systems that actively build and sustain a certain identity in time—conceived as physical separation from the environment (Maturana, 1975; Varela, 1979; Maturana and Varela, 1980/2012). Autopoietic systems are far-from-equilibrium systems that must counterbalance the natural entropic trends by remaining energetically and materially open, regulating their interactions with the environment without losing their organizational individuality (Di Paolo, 2005). Autopoietic systems are grounded in two basic processes—self-production and self-distinction—that are dialectically articulated in the enactive theory. Processes of self-production (also called self-maintenance) are those that contribute to incorporating elements and resources from the environment to build the structures that are necessary to maintain the viability of the organism. Processes of self-distinction, on the other hand, are those that topologically distinguish the organism from its associated environment. A cell is a typical example. The membrane is built and maintained by the cell itself through its metabolic activity (i.e., self-production) and at the same time, the membrane is responsible for separating the interior and the exterior of the organism (i.e., self-distinction). As self-distinction processes get stronger (e.g., less permeable membrane), the exchanges of matter and energy with the environment are reduced, reducing also its self-maintenance capacities. Meanwhile, self-production is facilitated by increasing the flux of matter and energy with the environment (e.g., making the membrane more permeable), that is, by going against self-distinction. This tension reflects the *precariousness* inherent in life. Unlike computationalist and connectivist views, from an enactive viewpoint precariousness of materiality is a necessary condition for a non-trivial definition of life and cognition in its broad sense. The very materiality of living beings makes life processes to be precarious, that is, to be likely to extinguish unless the self-organization of the living being actively sustains them. Therefore, the organism is in an active self-individuation process to perpetuate its own identity, a process that is grounded in the basic tension between participation and distinction (Kyselo, 2014), between interaction and constitution (De Jaegher and Froese, 2009).

In order to maintain its viability, the living organism is able to create and follow its own norms (or accept norms proposed by others). Indeed, the organism not only interacts reciprocally with the environment (e.g., as the sand that is moved by the wind and impacts the obstacles that it finds in its way), but it is also able to actively and adaptively regulate this interaction according to certain norms. In this regulatory asymmetry resides the *agency* of the organism (Barandiaran et al., 2009). Within this framework, agency is the capacity to discriminate between what is favorable or desfavorable for the viability of the organism, by dialectically resolving the basic tension between the two fundamental processes of the living, namely self-production and self-distinction. In this way, the organism acquires a perspective on the world, a sense of concern and caring (Colombetti, 2014). The environment is never neutral or inert, but it is affectively valenced according to the organismic needs and norms. Events do matter to the organism. This affective lack of indifference is the precondition for any form of cognition.

The *autonomy* of the organism is instantiated by a set of operationally closed processes, that is, by a set of intertwined processes that are mutually enabling. In an autonomous system, as explained earlier, the network of processes that is operationally closed is also precarious, i.e., it requires exchanges with the environment to endure (e.g., sunlight, oxygen, and nutrients). Importantly, the enactive concept of autonomy involves an active process that allows the organism to build its own identity through meaningful and adaptive interactions with its associated milieu (Varela, 1997; Di Paolo, 2005). In addition to the identity of the whole organism, the technical concept of autonomy allows to define different domains of organization that are co-instantiated in the same individual. For instance, a cell in a multicellular organism, the metabolic system of an animal, sensorimotor habits in mammals, or relational patterns in humans may be partly autonomous. These domains, which can be viewed as partially decoupled systems, influence, enable, and constrain each other but maintain certain autonomy and intrinsic normativities. In consequence, we can speak of different domains of normativities and semi-Minskian agencies and identities that co-exist in the same organism (Minsky, 1988). As we will explain below, this is relevant for elaborating an enactive conception of mental disorders because a usually neglected aspect of normativity is that, as a result of multiple norms that arise simultaneously, it is inherently tensioned.

Based on the notion of autonomy, the enactive framework interprets human bodies as involving three intermingled dimensions: organic, sensorimotor and intersubjective (Thompson and Varela, 2001). Recently, the linguistic dimension has also been proposed as an additional layer displaying autonomy and agency (Di Paolo et al., 2018). The organic dimension of embodiment corresponds to biological autonomy and it is shared with other living organisms. It includes a variety of interrelated processes and cycles such as metabolism, immune networks or hormonal regulations, which occur through fluxes of matter and energy with the environment. The sensorimotor activity of human bodies can also develop autonomy by generating a network of intertwined behavioral patterns that is precarious and operationally closed. This domain includes self-induced neural activity and a variety of cycles and feedback mechanisms that underlie the nervous system (Di Paolo et al., 2017). Habits manifest the autonomy that arises through sensorimotor coordination and perception-action loops, showing a form of autonomy that is dependent but partly decoupled from the biological one (Barandiaran and Di Paolo, 2014). In addition to the organic and sensorimotor dimensions of embodiment, social encounters give rise to another form of autonomy (De Jaegher and Di Paolo, 2007). Relational patterns, social norms, or cultural narratives, emerging from individual sensorimotor activity, can generate a life of their own and deeply influence personal practices through diverse looping effects and circular processes (Egbert and Barandiaran, 2014; Barandiaran, 2017; Fuchs, 2020). In the enactive framework, meaningful social interactions are described as participatory sense-making processes (De Jaegher and Di Paolo, 2007). As a result of interpersonal coordinations, a relational autonomy emerges that, without erasing individual autonomies, enables possibilities of action and meaning that were not accessible to individuals on their own. As a consequence, from an enactive perspective, human bodies are constituted by multi-scale dynamic processes involving a variety of organizational levels that are deeply enmeshed. This formulation overcomes traditional dualisms between mind and body, or between physiology and psychology in the formulations of mental disorders (de Haan, 2020).

The enactive framework emphasizes the necessity of investigating the interactions between processes at different domains taking always into account that they are part of integrated wholes as socially situated human bodies. In fact, the complex entanglement of processes that emerges in living bodies do not interact in a smooth and harmonious manner. There are several normativities at play simultaneously that generate conflicts and tension (e.g., “bad” habits and addictions that go against organic norms). In order to better understand how tension is created, transformed and released in human bodies, and how these processes can go astray in certain cases, in what follows, we will rely on some concepts and ideas from the philosophy of individuation proposed by philosopher Simondon (1958/2020).

## Simondon’s philosophy of individuation

The ontology that underlies the enactive framework is processual, relational, and holistic (Thompson and Varela, 2001). For this reason, Simondon’s ontogenetic metaphysics (1958/2020) is particularly useful to describe certain aspects of sense-making and mental disorders. As introduced before, Simondon coincides with the enactive endeavor of describing human beings (and other living beings in general) as unfinished entities involved in an ongoing process of self-individuation and becoming (Di Paolo, 2021). He invites us to think of the genetic process by which individuals come to being, their persistence, and their transformations instead of focusing only on finished, static, and self-standing entities. While the ultimate reality is defined in terms of processes, the individual is seen as an abstraction, a momentary and transitional state in the individuation process, an entity that is never fully constituted. The Simondonian ontology is also relational, which implies that relations are not merely links between relata that have a previous or independent existence, but relations are contemporary with the terms they relate and are, thus, constitutive of beings. Our goal is not to present his rich philosophical system in any systematic and exhaustive manner. For present purposes, it suffices to analyze some common points with the enactive perspective and present some concepts and ideas that can contribute to an enactive interpretation of mental disorders and symptoms^4^.

Simondon invites us to think of the genetic process by which individuals come to being instead of focusing only on finished and constituted entities (either physical, organic, psychic, or collective). The classical example of physical individuation is crystallization. Simondon claimed that to understand the constituted crystal, we should examine the crystallization process, that is, the phase transition from a supersaturated liquid solution to a crystallized solid structure. The supersaturated solution is in a metastable state, i.e., it shows only a limited degree of stability due to multiple tensions (e.g., forces among molecules in diverse directions). However, the system cannot release these tensions by itself to increase its stability. It requires a trigger. Hence, these tensions lead the system to a state with high sensitivity to external perturbations, i.e., tensions open potentialities for future transformations. Simondon calls the *preindividual* to the field of potentialities for change that transcends and extends the actual individual. Depending on the tensioned state of the liquid, the perturbation, and the environmental conditions, the phase transition will generate a concrete crystal with a certain size and crystallographic structure. The crystal is in another metastable state, but with lower potential energy and less tension than the initial oversaturated solution. At the same time, this process will reduce the preindividual load, the field of future potentialities of the system. Individuation is thus a process of generating structures or functionally distinct metastable states by transforming tension.

In contrast to physical individuation, individuation of the living does not take place in one shot or by combination of free-floating elements, but entails a progressive process. In the case of living organisms, self-individuating activity can be conceived as occurring in steps, each step transforming a certain amount of tension—but without exhausting all the tension and potentialities in the process. Living beings not only transform and release tension, they must also regenerate it in order to keep the system metastable and malleable (i.e., alive). They must actively interact with the environment to create and transform tension, exchanging matter and energy, and opening potentialities for future transformation. The metastable character of living beings is due to the preservation of a preindividual load, that is, a certain charge of potential energy that exceeds their organizational structure. According to Simondon, the self-individuation activity of the organism and, subsequently, sense-making operates through transitions between metastable states (Di Paolo et al., 2017).

Metastability in physics is defined as an energetic state of a dynamical system that does not correspond to the state of least energy. It is often related to conditions of unstable equilibrium and available potential energy. Through this concept, we want to highlight the malleable, adaptive and dynamical character of human bodies in relation to the notion of tension, that is, their potential for transformation. If there is no tension, there are no potentialities and there is no drive for individuation. In other words, stable equilibrium implies death. One way in which humans maintain those potentials open for future transformations is by keeping the system open to new domains of interactions with the environment in a process that is comparable with biological neotenization (Di Paolo, 2021). This implies slowing down biological development, which gives room to more sophisticated forms of cognitive functions. The biological vulnerability of humans in early life would not be possible without a high level of sociality. Conversely, that biological vulnerability opens domains of problematization (i.e., tensions) in the collective, giving rise to more complex social interactions. In this way, renewal of tension in humans takes place in the form of participation in the collective, learning new skills, engaging in intersubjective dialogue, or participating in a variety of groups.

In fact, in addition to physical individuation (generating material things) and living individuation (giving rise to biological organisms), Simondon also describes psychic individuation (which gives rise to perceptions, actions, emotions, memories, thoughts and so on) and collective individuation (which results in values, institutions, language, science, religions, or artistic works). Simondon describes psychic individuation as a way of solving a certain problem or tension in a higher dimension because it cannot be solved through a living individuation. It is important to remark that psychic individuation and collective individuation are tightly linked, although they do not necessarily happen simultaneously. Perception is clearly influenced by culture and social aspects, and at the same time collective individuation processes do depend on individual perceptions and actions.

This idea is captured by Simondon’s concept of *transindividuality* and it refers to the fact that our shared affects and coordinated behavior imply the possibility of participating in the other’s individuation and becoming (Heredia, 2015). Transindividuality, thus, implies participation in and modulation of others’ potential for change, that is, the modulation in the anticipatory character of other’s sense-making, opening potentialities for new meanings and actions that were not available to individuals on their own. Moreover, what this perspective questions is the idea that the two selves are fully individuated and self-acting and self-containing prior to intersubjective participation and interaction. In other words, interpersonal participation is not just something we as fully constituted subjects do, but something that constitutes us, like sensorimotor and organic processes do. This implies that in addition to viewing how the relational domain emerges from complex and dynamic causal interactions between individuals, we should also examine the global to local processes by which individuals individuate from the relational domain. In this way, gaining awareness of the degree of participation of the therapist in the individuation of the patient may open up more or less potentialities for co-transformation and change.

In other words, for Simondon, the individual is not the starting point of his investigations, but an effect of a continuous activity. Transindividuality is not the opposed relation between two already constituted terms, individuals and collectivities, but it articulates the relation of the individual with itself, with other individuals and the relation between different collectivities. This possibility arises due to the preindividual of each individual, the load of potentialities, which remains unindividuates and is partly shared with others. The most clear example of the transindividual is language. Language is an open system that changes with its use. It is not only the effect of individual behaviors, but also constitutes individuals by establishing a set of relations and structures that pre-configure the individual psyche. The transindividual character of humans thus refers to the inevitable participation in a variety of collective habits, norms and systems of value. Just like many different autonomous semi-Minskians agencies constitute and co-exist within the individual, which may pull the system in different directions by exerting opposing normative demands, a person also belongs to different social groups, whose norms may be in contradiction with each other. The participation in the social, then, is not univocal or homogeneous either, but tensioned, situated and highly dynamic.

The enactive approach and Simondon are indirectly related through the field of cybernetics, and their attempt of extending concepts and ideas from mathematics and engineering (control, stability, circularity, information) to biology and cognition. Enactive cognitive science can be considered as a branch of second-order cybernetics (Froese, 2010), while Simondon established dialogues with authors of the first wave of cybernetics (e.g., Wiener) and tried to go beyond their notions of adaptation and information^5^. We find strong similarities between the simondonian philosophy of individuation and the enactive approach. First, the preindividual, that is, the excess of virtuality inherent to the actual individual, is a fundamental aspect of sense-making and *adaptivity* (Di Paolo, 2005). Adaptivity refers to the capacity of determining future potential trajectories (shaped by the current organizational structure), evaluating them according to the norms of the organism and its viability conditions, and regulating the interaction with the environment accordingly (Froese and Ziemke, 2009). The living organism is one that can evaluate not only its actual states, but also its tendencies and potential directions. Being sensitive to potential challenges and risks, it can regulate its activity and its interactions to avoid, limit or compensate for potential challenges to its viability conditions. Second, affectivity plays a crucial role in simondonian philosophy and it is tightly linked to collective individuation and participatory processes. Several interpretations of the simondonian perspective on affects point to their function of ordering disparate affective forces in a coherent positive-negative, pleasant-unpleasant, proximal-distal polar axis (Massumi, 1995; Heredia, 2012; Wrbouschek and Slunecko, 2021). Affects thus play a crucial role in relating the individual with its preindividual, i.e., with the set of available potentialities (Heredia, 2012). In this way, affects and emotions reflect also the transindividual character of humans. While affects manifest the relation of the individual with itself, emotions are collective stabilizations of affects and reflect the unity of the psychosocial activity. Affects and emotions are not fundamentally distinct, but emotions are collective stabilizations of affects. They face the problematic of the subject, that is, the fact that the subject is inherently individual and collective at the same time and, as such, it needs to appeal to the collective to self-individuate, that is, to feel oneself (Tucker, 2022). In the same line, the enactive notion of adaptivity, as a form of responding as a whole to virtual trajectories of the system, is inherently affective, not only in virtue of distinguishing between positive and negative tendencies, but also in virtue of integrating the disparate affective forces, sometimes responding to different normative domains. Third, the perspective on metastability aptly describes the dynamics of sense-making. Indeed, sense-making aims to capture the transitions between dynamic states, rather than stationary states of functioning—what traditionally have been called “mental states”. Fourth, the self-organization and continuous renewal of tension that is present in organic individuation resonates with the enactive concept of autonomy. Living, psychic and collective (plus physical) individuation processes are deeply intertwined and follow similar logics, in line with the intermingled dimensions of embodiment (organic, sensorimotor, and intersubjective) based on the enactive autonomy and the life-mind continuity thesis. Therefore, without entering in depth in the philosophy of Simondon, we can see that some of his concepts and ideas, while being compatible with the enactive approach, offer a novel perspective or a view from a different angle that can be inspiring. Such a different point of view can complement the enactive theoretical framework and will allow us to better describe certain processes related to mental disorders.

This processual and relational perspective based on metastability, tension and individuation processes encourages a significant change from approaches to life based on homeostasis (or allostasis). Homeostasis refers to the optimal steady state of an organism, where internal variables are maintained in a certain range that favors the viability of the organism. Interactions with the environment are seen as perturbations altering this state that the organism must compensate for. In contrast with this static view, the notion of allostasis corresponds to stability through change, including the capacity of the organism to predict future events and produce changes in an anticipative manner. These notions, together with feedback loops, have been very successful in physiology, and they are still widely employed in clinical practice (e.g., to treat hormonal disbalances). However, and despite homeostasis, allostasis and feedback loops seem adequate to describe the regulation of local variables in a flat chain or short timescales, they cannot capture the full complexity of the organization of living beings and their regulation mechanisms (Bich et al., 2020; Bardin and Ferrari, 2022). This is especially problematic in long-term processes. Therefore, the extrapolation of these concepts coined in physiology to explain and treat mental disorders have strong limitations. Arguably, growth and transformation are fundamental for self-individuation of living beings, not only subsistence. This feature requires adopting a historical path-dependent perspective. While the homeostatic and allostatic views emphasize stability (constant or through changes) and relegate regulatory mechanisms to balance (potentially) altered variables, the metastable perspective would emphasize transformation, claiming that there is no stable state to which the organism should go back. Instead, progressive states of partial, momentary and precarious stability are sustained as a result of opposing regulatory demands. If an homeostatic stable state can be described, it is a local homeostasis sustained by a variety of forces and tendencies that pull the system in different directions. That is, temporarily identifiable stable structures that emerge from the integration of dynamical processes in a given localized domain^6^

The transformative character of the living organism, that is, the character of being directed toward virtuality, is a manifestation of its temporal asymmetry. This implies that living organisms are not only temporal but also historical (Di Paolo et al., 2021). Living processes do not only compensate for each (potential) perturbation. Each interaction with the environment, each transition between metastable states, can alter the potentialities available for the future (the preindividual). Neural plasticity, development, incorporation of new habits, personality, intersubjective relationships and so on are historical because they allow for cumulative change and transformation. Every (step of) individuation reduces the variety of potential trajectories to one actual state while opening up new paths for future individuation. Accordingly, sense-making does not occur anew, but strongly depends on the particular history of organism-environment couplings and their mutually constructed dynamical dependencies. Those dependencies may encompass processes along different timescales, basic processes being entrained by larger-scale processes. This allows us to identify a multiplicity of temporal scales in sense-making. For instance, identifying a movement not only as a mere movement, but as an intentional act with diverse possibilities for meaning depending on the scale and the context (e.g., shaking hands between two politicians). This historicity of sense-making is what confers a particular, concrete, and unique perspective to the individual. As a result, investigating particular embodied and situated experiences is fundamental in order to account for the variability and diversity in mental disorders and human life in general.

In sum, from the enactive and simondonian perspective developed here, living beings (and humans) are open, indeterminate, and historical beings in a constant process of self-individuation. In addition to its current state, the individual also involves its history, with the results of cumulative individuation processes and partial tension resolutions, and the set of preindividual potentialities that are still open and keep it alive.

## Mental disorders as disorders of sense-making

A core tenet of the enactive approach is that the mind is not located in the brain, but emerges from the embodied interaction of the agent with its environment. Mental disorders, then, cannot be reduced to direct consequences of brain disorders, nor to mere social constructs. They should be understood in terms of non-linear, highly complex interactions between organic, sensorimotor, and intersubjective transactions with the environment (de Haan, 2020). Moreover, the entanglement of different dimensions of embodiment implies that there is no privileged description or dimension to define pathology (Mc Gann and Cummins, 2013). Indeed, the categorical distinction between somatic and mental disorders loses its grounds in this framework. All health problems may be seen as disorders of sense-making in a broad sense as they all involve organic, sensorimotor and intersubjective aspects (even if in different degrees or contributions). However, this is not the same as claiming that every domain has the same importance in every case. Some disorders may benefit from an intervention at the physiological level (e.g., pharmacological), others may improve with a therapy at the psychological domain (e.g., psychotherapy), and other conditions may require influences at different dimensions (e.g., socio-community interventions). And a combination of interventions at diverse dimensions simultaneously may be useful as well. It is important to notice that interventions in a concrete realm often have strong influences in other dimensions. A physical intervention (e.g., a surgery) is not only affecting the organic body. A surgery may have a profound impact in the sensorimotor and social domains, as it may involve sensorimotor limitations and changes in the social environment (e.g., being at a hospital, enhanced social support) that can modify the way of life of patients. At the same time, a sensorimotor intervention may help without directly affecting physiological conditions. In patients with arterial disease in the lower limbs, walking therapy can contribute to reduce pain, increase walking capacities and improve their life—without directly intervening in the arteries. The success of walking therapy, moreover, depends on the trainer and her effort, showing again the deep interrelation among dimensions of embodiment (Mol, 2002).

The enactive framework and the ideas from the philosophy of Simondon presented here allow us to elaborate on what has already been said on disorders of sense-making. We want to focus on five aspects that are relevant for our purposes: temporality, the relational aspect of sense-making, normativity, tension and participation.

### Sense-making is a process: dynamics and historicity

The processual and relational nature of the enactive approach and the philosophy of individuation developed by Simondon encourages us to shift the perspective on mental disorders from states-like entities occurring in passive individuals toward dynamical and interactive processes enacted by socially situated embodied agents. Accordingly, mental disorders should not be described (only) in terms of their structural, static and individualistic aspects, but as *processes* that individuals undergo in interaction with their associated sociomaterial environment. Mental disorders have an inexorable temporal dimension and identifiable time courses, where we can distinguish stages, relapses, and recovery tendencies (e.g., panic attacks are more rapid and acute than depressive processes). From our perspective, the temporal course of mental disorders should be seen as intrinsic to what they are.

We suggest that any classificatory system—either the RDoC project (Cuthbert, 2014), the ICD-11 (WHO, 2014, 2020) or the DSM system (American Psychiatric Association, 2013)—would benefit from paying more attention to the temporal dimension in their definitions, rather than focusing mostly on the set of symptoms—what has been called the “homeostatic property cluster” (Kendler, 2016). Indeed, many subclinical disorders and affective episodes can be viewed as processes that a person undergoes rather than fixed and static properties (e.g., stress, anxiety, or non-chronic depression). These experiences can become clinical disorders if they surpass a certain intensity threshold, extend for a certain period of time, or appear with a determined frequency. The DSM-5 already considers frequency and duration of observable symptoms in establishing the severity of certain pathologies (American Psychiatric Association, 2013). However, beyond frequency and duration, we claim that mental disorders manifest identifiable dynamical patterns of development and recovery which should be considered as inherent of what they are. For instance, depression shows emotional inertia toward a negative mood sustained by feedback loops, which favor profound relapses that extend in time (Demic and Cheng, 2014; Hayes et al., 2015). Conversely, anxiety disorders show larger mood variability and instability (Lamers et al., 2018). In turn, schizophrenic patients may show long periods of prodromal phase before active psychotic episodes appear and also long periods of remission sometimes accompanied by post-psychotic depression (Moritz et al., 2019). The intrinsic temporality of each disorder is different and crucial to understand many of them. As a result, a process perspective on mental disorders would allow us to move away from the relatively fixed and stable categories and the reificating tendency of looking at mental disorders as natural kinds. The enactive framework would maintain a realist perspective on mental disorders without describing itself to substantialism or essentialism (Zachar, 2000; Kincaid and Sullivan, 2014; de Haan, 2020).

This dynamical perspective goes along with the change process research in psychotherapy (Elliott, 2010). Attending to the time-course of the emergence, persistence, and decay of certain psychopathologies allows identifying the interconnection between short-term and long-term effects of therapeutic changes and recoveries (Hayes et al., 2007, 2015; Schiepek et al., 2017). Indeed, the non-linearity of therapeutic changes, — i.e., the more or less abrupt transitions between relatively stable phases along the therapeutic process (Gelo and Salvatore, 2016)—is very likely to be influenced by the inherent temporal rhythm of the disorder itself. This process perspective on mental disorders may allow us to build individualized models of disordered patterns and may facilitate a transdiagnostic understanding of psychopathology and the therapeutic process (Salvatore et al., 2015; Nelson et al., 2017).

In addition to incorporating dynamical aspects, we also suggest viewing therapeutic processes and mental disorders as path-dependent. This means that mental disorders are cumulative and non-homomeric processes—i.e., they can be described in phases [building on the typology of processes developed in Seibt (2004)]. A relapse in an addiction rehabilitation process does not place the patient in the same state as in the beginning. Mental disorders (and life in general) involve irreversible processes where there is a progressive cumulation of changes. In addictions, for instance, a relapse typically entails a decay in the confidence of self-capacities to attain the therapeutic goals, involving a negative feedback loop that hampers future change processes. Being so, the therapeutic strategy should not only rely on the diagnosis and the current symptomatology of the patient, but should consider the trajectory of the person, how the disorder has evolved, and the history of interpersonal interactions that have led the person to the current situation. This is indeed a common practice in most forms of psychotherapy. What is missing, we believe, is the theoretical, epistemological and even ontological description of mental disorders as path-dependent, heteromeric, recurrent, and non-linear processes. This perspective allows us to move away from the static and reifying approach promoted by current classificatory systems.

### Sense-making is relational: adaptation vs. adaptivity

The downplay of the dynamical and historical nature of mental processes is likely to be related to the popular view of life and health in terms of adaptation and homeostasis. The homeostatic view presupposes an optimal stationary state to which the organism should return, usually based on statistical normality (Boorse, 1977). When the interaction with the environment is taken into account, health and disease are typically interpreted in terms of *adaptation*, that is, the ability of the organism to flexibly adjust to different internal and external conditions. As a result, pathological dysfunctions are seen as failures in fulfilling the adaptive function (e.g., Selye, 1975; Kovács, 1998; Beck et al., 2021).

However, we should distinguish adaptation from the notion of *adaptivity* developed within the enactive framework (Di Paolo, 2005). While adaptation implies a changing environment to which the organism should adjust, adaptivity has to do with the ability of anticipating potentially undesirable trajectories in the organism-environment couplings and regulating the interaction accordingly. There is a difference between coping with the environment in a purely reactive manner and modifying the coupling itself or generating supportive environments. While adaptation implies intervention to counterbalance external changes, minimizing its effects in order to maintain a stable internal milieu, adaptive regulation implies maintaining certain internal flexibility, regulating the interaction with the external world, or even intervening in the environment. To put it succinctly, adaptivity implies taking advantage of change rather than merely preventing it (Menatti et al., 2022).

There are convincing reasons to avoid the adaptation criterion in the definition of mental disorders. The main concern is that, as repeatedly pointed out by the anti-psychiatry movement (Cooper, 1967/2013) and more recently by the neurodivergence movement (Dyck and Russell, 2020), the adaptation criterion can promote pathologizing individual suffering heavily influenced by social structures. Indeed, one can be maladaptive without being pathological and pathological without being maladaptive, as in cases of social success (e.g., in academic work) at the expense of stress and anxiety. Similarly, contestation or lack of adaptation to authoritative social structures should not be regarded as pathological. In addition, if we assume the relational ontology proposed by the enactive approach, there is no fully-constituted organism that needs to adapt to an *a priori* constituted environment, but a mutual co-constitution of organism and environment. The adaptation criterion oversees the mutual transformation and feedback loops between organism and environment, sometimes formulated as the niche-construction activity of the organism which modifies the sociomaterial environment and constraints, bootstraps, and modulates its own behavior (Laland and O’brien, 2011). If we assume the active role played by the individual in anticipating and modifying its coupling with the environment, then health is not only related to the responsive capacity of the individual, but with its preventive capacities. A similar shift from adaptation to adaptivity can be seen in discourses promoting a preventive medicine over treatment-based medicine (Larson, 1999; Menatti et al., 2022).

As a result, adaptation may be neither necessary nor sufficient to define mental disorders. An organism can be structurally non-adapted in a concrete moment but organizationally adaptive, that is, it can manifest adaptive behavior even if it is not properly adjusted to the actual situation. Adaptivity also operates over virtuality, at the level of the preindividual, by anticipating and modifying the field of potentialities of becoming. If there is maladaptation in certain psychopathologies, we suggest it may be in virtue of a lack of coherence in the individuation process between the actual and the potential, between the current state and virtual possibilities for the future. This might be related to an overly rigid or overly flexible sense-making that does not integrate diverse norms and needs that traverse the individual (we will unpack this idea in the next section).

To put it succinctly, the enactive approach advocates for a processual and relational perspective on pathologies that is sensitive to the historical aspect of living organisms. Accordingly, the relationality of mental disorders should not be understood as a relation between fully constituted and static individuals and environments, but as dynamically changing and mutually constraining processes that are path-dependent. The proposal is to look at the (pathological) organism-environment relationship as an emergent product of the self-individuation process of the individual.

### Sense-making involves managing multiple norms

A classical way of understanding pathology has been as an impairment of building and following one’s own norms. This formulation—first made by Canguilhem (1966/2012) and more recently endorsed by Nielsen and Ward (2020)—overcomes the limitations of defining health and disease in terms of external norms (e.g., social values, statistical normality). From this perspective, psychopathology would imply a systematic or structural break of the functional norms and values of the individual. This form of naturalized normativity, however, bears the risk of understanding living processes in overly functionalist terms (de Haan, 2021). Simondon’s distinction between norms and values is relevant here (see also Di Paolo and De Jaegher, 2022). While norms can be understood as referring to the function of an individuated (stationary) system, values refer to the forces that drive individuation processes, which are operationally (rather than structurally) defined and, as such, can be seen as the genesis of norms. Values thus correspond to acts and operations occurring at the transitions from one metastable state to another, from one logic of functioning to another. Accordingly, they do not follow a close system of norms. Instead, values express a relation between current and potential states. Simondon puts forward an axiology that gives room for the invention and generation of new norms^7^. Following this perspective, we point out that normativity does not exist in the abstract in the form of *a priori* set of rules the organism must follow. The whole system instantiates a norm of self-maintenance by being operationally closed, but the norm is not in the mechanism or purposes of the system. Rather, norms should be understood as emergent phenomena that are linked to sets of possibilities for action and change in the system; that is, norms are situated and concrete actualizations (Di Paolo et al., 2010). The norms of the system should be understood as moment-to-moment evaluations of the matrix of possible actions, rather than as general and abstract rules that particular actions must follow. Norms are explored through meaningful interactions with the environment that generate tensions, and in permanent negotiation to transform tension and minimize conflicts. Those matrices are balanced according to the self-individuation tendencies of the organism, its actual state and potentials, and long-term goals. They provide directionality to the individuation of the system while encompassing diverse time-scales.

Moreover, the enactive approach wants to acknowledge the multiplicity of entangled normativities of the human form of life. Organic self-maintenance, we argue, should not be seen as the reference rule to which all norms subsume. Indeed, many human behaviors, which should not be seen as pathological, go against basic or organic norms, by postposing certain biological demands in favor of the attainment of longer term goals. If we assume the enactive proposal of looking at distributed normative semi-Minskian agencies, then there would not be a static, integrated, and coherent norm (or sets of norms) that drives the organism—and whose breakdown would indicate pathology. Accordingly, health and pathology should be distinguished in terms of the moment by moment emergent clusters of norms that evaluate the current situation the organism is embedded in. A clear example is fasting, which can go against a basic organic norm of nutrition if it extends for too long. Nevertheless, fasting by itself does not indicate pathology, nor is pathology defined in terms of the underlying causes that generate fasting behavior. In order to consider a certain behavior a pathological symptom, we need to look at the distribution of the whole network of norms. In anorexia, for instance, we can identify two sets of norms that are in conflict; namely, the normativity of the body as a living organism with its nutritional needs and the normativity of the body as an object of self-concept which is strongly influenced by social norms. Fasting can be motivated by religious beliefs (i.e., a social norm) as well, and can be neutral, beneficial or a source of pathology depending on the extension, the previous health situation and the activities and responsibilities during the process (e.g., physical work). Pathology, thus, is not located in the breakdown of a single norm but in the network of norms that traverse the individual.

Addictions represent another example of the simultaneous emergence of multiple normativities, which are often in tension or generate internal conflict. For instance, smoking, being a sensorimotor habit that damages the respiratory system, creates a tension between normativities related to sensorimotor and organic dimensions of embodiment. In the example of smoking, we can also find conflictive norms at the organic dimension of embodiment, as it is detrimental for the respiratory system but the need of nicotine can become an established regulatory mechanism at the organic level, whose breakdown triggers abstinence symptoms. The sensorimotor act of smoking already constitutes a self-sustained behavioral pattern that develops its own autonomy (Ramírez-Vizcaya and Froese, 2019). This is why people in the process of quitting try to substitute the habit by keeping the sensorimotor pattern, usually bringing something to the mouth (e.g., eating candies). At the intersubjective domain, smoking might help the socialization process (e.g., generating a sense of belonging to a social group in teenagers) or it can be a resource to regulate interpersonal interactions (e.g., leaving momentarily social encounters or atmospheres that are uncomfortable with the excuse of smoking). All three dimensions of embodiment and their distribution of norms should be considered in our understanding of addictions. Looking at the network of sensorimotor schemes and at the interrelated intersubjective realm where the habit is immersed can be very useful to recognize situations that entail the danger of smoking (e.g., excessive workload, working environment, family meetings, or interpersonal encounters with some friends).

In sum, we advocate for a perspective on normativity based on the continuous integration of a variety of norms—local, dynamic and focused toward the future—that can be in conflict or show some degree of incompatibility. In order to better investigate this process we develop the notions of tension and individuation processes in the next section.

### Life and sense-making are driven by tension

Disease and illness, including mental disorders, are usually associated with tension and imbalance, often involving a lack or an excess (e.g., an excess of meaning in psychotic experiences, lack of certain neurotransmitters in depression), a dysfunction or an overfunction (e.g., dysfunction of the theory of mind module in autism, dysfunction of self-control mechanisms in addictions, excessive self-reflection in OCD).

From the enactive-simondonian perspective displayed here, tension is not exclusive to disease and illness, but ubiquitous in living bodies. Through the interaction with its associated environment, a living body is in a permanent, although dynamic, situation of tension and critical imbalance that drives self-individuation. As a consequence, tensions should be considered as inherent aspects of life, rather than as negative situations arising in a broken, out of balance or malfunctioning body. Illustrative examples are the necessity of being in contact with all sorts of pathogens in early phases of life to build the immune system (Brodin and Davis, 2017), the need of a certain degree of affective variability to flexibly attune to changing situational demands (Chan et al., 2016), and the need of interactive breakdowns and recoveries to mutually readjust in therapeutic processes (Safran et al., 2001; García and Di Paolo, 2018). Living beings are precarious, which implies that all living processes, taken in isolation, would lead the system to its death unless the whole self-organization of the living compensates for them.

In order to better understand the dynamics of tension creation and transformation it seems necessary to investigate how tension emerges. Tension appears when multiple regulatory demands arise simultaneously in a non-harmonious manner. There are a variety of norms that can arise simultaneously, and some of them can be completely or partially incompatible or mutually exclusive with each other. In human bodies, tensions are present at different organizational levels, both intra-level and inter-level. They arise at the organic level, for instance, between hormones with antagonistic effects (e.g., insulin and glucagon, parathyroid hormone and calcitonin), between sympathetic and parasympathetic nervous activity, or between cells with opposing roles (e.g., osteoclasts and osteoblasts). Likewise, the sensorimotor dimension displays tension between flexor and extensor musculature, or among the variety of actions available for an active embodied agent with a particular sensorimotor repertoire in a certain context. At the intersubjective dimension, we also find a deep tension between individual and relational needs that permeates all social interactions (Kyselo, 2014) as well as between behavioral demands of different social systems one may belong to Di Paolo et al. (2018). Moreover, tension can also emerge among conflicting normativies at different dimensions, as demonstrated by the difficulties to eliminate “bad” habits and harmful addictions. Going back to the example of smoking introduced earlier, the difficulty of changing habits is a clear example of tension between dimensions of embodiment, and also between pre-reflective and reflective activity (Gallagher, 2012). The reflective intention to quit smoking is necessary, but not always sufficient to overcome the pre-reflective impulse to maintain the habit^8^. Indeed, metastability and tension pervade all domains of reality and describe individuation processes in general. For instance, the dynamics of an argument between two people can also be studied in terms of metastability where small interventions might lead a couple to split up, to reach a partial consensus or to keep arguing (Veitas and Weinbaum, 2017).

Perhaps the best example of tension in the human body is pain, which clearly shows how deeply enmeshed the three dimensions of embodiment are (Stilwell and Harman, 2019; Coninx and Stilwell, 2021; Miyahara, 2021). Pain, for instance, is a fundamental process of embodied experience that can both affect and be affected by organic (e.g., tissue damage), sensorimotor (e.g., physical activity) and intersubjective processes (e.g., social support). Pain is also considered a drive for change (Quintner et al., 2008), that is, a trigger to modify the current set of tensions of the living body.

Despite tension being an intrinsic part of biological and mental processes, how tension is created, transformed and released can shed light on symptoms and mental disorders. From this perspective, some mental disorders can be interpreted as involving a metastable state with an excess of tension or with difficulties to trigger an individuation process that transforms and releases tension. For instance, phobias, Obsessive Compulsive Disorder (OCD) or Post-Traumatic Stress Disorder (PTSD) can be seen as generating an amount of tension that is excessive and not in accordance with external situations. These conditions may benefit from a gradual exposure that aims at reducing the sensitivity to situations that trigger excessive tension (Craske et al., 2014). In the same vein, addictions often require withdrawing from certain contexts that generate excessive tension and favor addictive behavior (a bar for an alcoholic, a mall for an addict to shopping or a group that facilitates addictive consumption or activity), and gradual exposure once they have developed skills to manage tension and better cope with these situations. In contrast with these conditions, Borderline Personality Disorder (BPD) can be interpreted as a blockage to transform tension. In this case, patients manifest difficulties in integrating contradictory aspects and tendencies and building a coherent narrative of the self (Fuchs, 2007). The amount of tension created in this case is not specially large, but the individual may not have the capacity to manage interpersonal difficulties (Zanarini et al., 2007). The impulsivity and lack of coherence of BPD behaviors are usually not attuned to the situation, and they often aggravate the tension they attempted to release or solve.

In this regard, affectivity plays a fundamental role in regulating the tensions of the living, being manifested in organic, sensorimotor and intersubjective domains of embodiment. As introduced before, sense-making is fundamentally affective insofar as the world is not indifferent to the individual, but affectively valenced (Colombetti and Thompson, 2008). Affectivity, in this context, integrates local tendencies, forces and diverse normativities in a coherent and integrated organismic attitude (Wrbouschek and Slunecko, 2021). It thus anticipates certain coherence in the individual-world coupling by operating over preindividual potentialities (Keating, 2019). Affects predispose the individual to certain interactions, closing or opening the field of potential trajectories for individuation. For instance, depression does not only imply being stuck in a sad mood, but also a general diminishment of the field of potentialities for action or *affordances* (de Haan et al., 2013), a diminishment of the sense of self or affective depersonalization (Aho, 2013), and impaired bodily coordination and affective participation with others (Fuchs, 2013a,b). Depression implies an atmosphere of emotional indifference, which hampers meaning-making and diminishes the sense of self that accompanies meaningful experiences. In other words, it curtails self-individuation by decreasing potentialities in one’s becoming. This decrease of future potentialities is directly captured by the concept of the preindividual proposed by Simondon.

### Sense-making is participatory

Sense-making, in the case of humans, is always and from the beginning an intersubjective and social enterprise (De Jaegher and Froese, 2009). Our cognitive capacities, the categories we employ for meaning making, our perceptive habits and affective valences are deeply constituted by the social environment we are embedded in and our history of social interactions. As a consequence, every mental disorder has an inexorable intersubjective dimension, which has generally been downplayed in psychiatric accounts of mental disorders.

From the enactive perspective, several examples have been raised highlighting the intersubjective character of mental disorders. Indeed, a large proportion of our engagements with others imply a bodily coordination or attunement with others—both in terms of vital rhythms and affects (Fuchs, 2005, 2013b). Autism, for instance, can be better understood as a disorder of the pre-reflective embodied engagement with others in face-to-face encounters than as a dysfunction in the “theory of the mind” or mindreading module in the brain (Gallagher, 2004; De Jaegher, 2013). Anxiety disorders may also result from complex interactions and looping effects between individual behavior and interpersonal responses (Glas, 2020). Although some character traits, such as attachment styles, may have a clear causal role in the emergence of anxiety disorders, individual traits do not fully explain the dynamical trajectory that leads a person to fall into a certain pathological state. In this case, the atmosphere at work-places, support of family and friends, and relational styles may contribute to the emergence of a panic attack.

For Simondon, anxiety is the result of a turn inward, an attempt to solve an affective tension alone, without participating in the social realm. Anxiety is central to understanding the relationship between the preindividual and the transindividual, and the connection between psychic and collective individuation. Anxiety emerges with the impossibility of resolving the problem of affectivity, that is, the incompatibility between the constituted individual and his pre-individual load. The excess of pre-individual potentialities requires the collective to actualize itself. There is a difficulty to integrate diverse affects—psychic individuation—because there is a lack of connection with the external world—collective individuation. In anxiety “emotion becomes amplified and internalized; the subject continues to be and operate an ongoing modification within itself, but without acting, without being inserted into or participating in an individuation” (Simondon, 1958/2020, p. 284). As explained earlier, emotions and psychic life in general, for Simondon (and for the enactive approach), cannot be reduced to private states of the individual and demand participation in the collective. The anxious subject tries to solve the affective problem looking only at itself, entering in a mode of solipsistic individuation doomed to fail. Anxiety is thus not only a consciousness of separation from the collective, but the impossibility of actualizing the potentialities within the individual. That is, an attempt of sense-making isolated from the intersubjective domain. In fact, anxiety and other symptoms show important differences among cultures (e.g., Kirmayer et al., 2011), which suggests that they can be influenced by implicit social learning.

To provide another example, schizophrenic delusions may emerge as feedback loops between biological, behavioral, and intersubjective factors (Fuchs, 2009; Van Duppen, 2017). Biological imbalances may contribute to social withdrawal that in turn leads to a misattunement with others, which feeds back onto the individual increasing their probability of undergoing a psychotic episode and delusions. Indeed, the embodied minimal self, which provides a coherent sense of self and is disturbed in delusional experience emerges from early social interactions and temporal attunements with others (Kyselo, 2014, 2016). Interpersonal interactions, thus, not only impose causal constraints to the emergence and persistence of mental disorders; they play a key constitutive role in their generation. In other words, we can say that the bearer of the pathology is not only the individual. Mental disorders are relational phenomena that stem from the dynamic interplay between individual and intersubjective interactions.

Accordingly, the enactive approach necessarily adopts a second-person perspective on psychotherapy (Galbusera and Fellin, 2014), where the therapeutic relationship is characterized as a social interaction based in resonance, engagement and mutual responsiveness. The therapist is no longer seen as a detached epistemic subject that approaches the patient as a passive object of knowledge. Instead the therapeutic process is seen as a dynamic process of participatory sense-making where both participants contribute to shared meanings and both are transformed by them (García, 2021). The transformative role of the therapeutic relationship is explained by this intersubjective character of humans, that is, the fact that our interpersonal engagements with others represent a form of co-individuation and co-constitution.

As a final remark, the entanglement between dimensions of embodiment, and the effect of interpersonal interaction in symptoms is particularly evident in placebo phenomena. Placebo effects are psychosocial interventions without direct physiological influence that have been shown to have positive therapeutic influences in a variety of conditions (e.g., Benedetti, 2020). One of the aspects that placebo research highlights is the impact of concrete social encounters in symptoms and health outcomes—for instance, between patient and practitioner (e.g., Kelley et al., 2009), or between parent and children (Czerniak et al., 2020). In line with enactive ideas, these social interactions have been interpreted as participatory sense-making processes (Arandia and Di Paolo, 2021). This can be illustrated by the open-label placebo experimental paradigm. In these settings, participants are given a placebo pill and are told that it is a placebo. Therefore, there is no deception and no uncertainty over whether there might be some kind of direct physiological influence at work. However, the interaction with the researcher or practitioner in these cases also includes some interventions that open possibilities for change, such as talking about “a novel mind-body treatment” (Carvalho et al., 2016, p. 2767), explaining scientific evidence that supports placebo effects (Kaptchuk et al., 2010) or emphasizing the importance of following the instructions to take the placebo pills as in usual treatments (Hoenemeyer et al., 2018). The performance of the practitioner has been shown to be relevant as well (e.g., Thompson et al., 2009), with variability in therapeutic outcomes among practitioners following the very same protocol (e.g., Kelley et al., 2009). In the same vein, the sociocultural context can also have deep influences on placebo phenomena (e.g., Kirmayer, 2011). Therefore, placebo phenomena reflect the transindividual proposed by Simondon—the deep entanglement between organic and intersubjective realms—while opening room for investigating their mutual influences in therapeutic practice.

## Discussion

The enactive framework presented here depicts human beings as permanently and actively building their own identity in a self-individuation process. Humans need to develop and renew goals, skills, potentialities, and tensions in order to maintain the coherence of their socially situated bodies while remaining open to further transformative interactions with the environment. This approach highlights processual and relational aspects of mental disorders emphasizing transformation and adaptivity over the static view promoted by homeostasis and adaptation-based health models. This view also highlights path-dependence of mental disorders taking into account accumulated past individuations and experiences. Self-individuation involves a historical and non-linear process full of complexity that advances by combining sudden abrupt transformations with more subtle and gradual changes, often overlapping individuation processes at multiple scales. Adopting the perspective on individuation proposed by Simondon, the renewal of tensions within and across biological, sensorimotor and intersubjective dimensions are considered as constitutives of human life. Living processes entail a dynamical regulation of distributed and situated norms along different dimensions of embodiment. These regulations take the forms of local gradients and trajectories whose integration leads to progressively more complex organism-environment structural couplings. Interpersonal interactions, in turn, are seen as constitutive of mental disorders, being involved in looping effects that modulate their emergence, persistence and decay.

Within this context, the interpretation of symptoms and therapeutic interventions should be based on knowledge of the whole organism, including temporal and relational aspects. Symptoms are no longer understood as compensatory mechanisms of going back to an homeostatic and relatively stable state, but mechanisms that do play regulatory roles when the whole organization is taken into account (Merleau-Ponty, 1945/2012). This view suggests interpreting symptoms as consequences of a complex tensioned network involving multiple normativities that demand further changes and individuations. We consider psychological symptoms as calls for individuation, as excessive tension that needs to be partially released in a meaningful and transformative way. In this sense, certain symptoms can be seen as manifestations of the preindividual load of the organism, its potentialities for change and transformation that, for some reason, are partially blocked. Accordingly, symptomatic behavior entails sensorimotor patterns that do not help transforming tensions but just allow a partial release—which might have short-term benefits but do not contribute to opening potentialities for long-term improvements. Instead, these behavioral patterns may reinforce themselves, diminishing the preindividual field of potentialities and favoring the generation of excessive tension in the future (e.g., in addictions). Despite their obvious negative effects at the experiential level, some symptoms may be seen as having positive value to the patient insofar as they demand transformation and change (i.e., opening potentialities, enhancing the preindividual) and preventing decline into a more debilitating state of affairs. Hence, symptomatic behavior should be seen from an integrative perspective that uncovers their meaning for the whole organism. As the three dimensions of embodiment are deeply intermingled, tension in one domain may reflect a problem in another domain, or offer possibilities to be released through another domain. For instance, a typical apathy in depression is not a symptom merely at the individual level, but involves changes in the patterns of interpersonal relatedness (Fuchs, 2009). A similar interpretation might be applicable to addictions. Beyond the tension between organic and sensorimotor norms, habits (including “bad” habits and addictions) may have a meaning when we zoom out to the social domain as the social regulatory function of smoking explained above. In this case, a sensorimotor habit may contribute to release tension that originated in the intersubjective realm. Taking into account the entanglement among dimensions of embodiment and the role of tension may facilitate developing more integrative interventions.

The implications of adopting a relational and process perspective on pathological symptoms and mental disorders are manifold. The simondonian and enactive approach presented here invites us to look at the temporal aspects of mental disorders, that is, as developmental processes that constitute the individual. In this sense, mental disorders should not be seen as separated from the individuation process. They are processes that an individual enacts in his/her functioning. Accordingly, mental disorders are not only suffered, but they are enacted (Svenaeus, 2022). This perspective also allows us to focus on change rather than on stability, that is to stress the role of phase transitions between stages, relapses, recoveries and moments of different intensity. This perspective contrasts with the biomedical paradigm, which aims at identifying and treating dysfunctional mechanisms following reductive methods that are constrained to objectively measurable physiological processes. The entanglement between physiological, sensorimotor/psychological and social processes, and the relevance of the interactions among these realms also contrasts with the biopsychosocial model (BPS). In practice, BPS tends to treat the three fields as independent, often assigning more importance to the biological and downplaying the interactions between the three domains. The target of the enactive approach, instead, is the whole organism, coupled with its associated sociomaterial environment and immersed in an ongoing individuation process. That is, it encompasses the entangled organic, sensorimotor and intersubjective dimensions of embodiment in a dynamic and transformative interaction with the environment.

As one can notice, the enactive approach shares some principles with systemic perspectives to psychotherapy (Minuchin, 1974/2018; Hardham, 1996). Building on insights from early cybernetics (Bateson, 1972/2000), systemic psychotherapies advocate for understanding every act or event as being a sign of the whole interpersonal/organizational/family system the individual is embedded in Minuchin (1974/2018). Symptoms are emergent wholes rather than mere individual activity and thus are manifestations of interactive patterns of the entire system. The enactive approach we are presenting here takes a systemic perspective in the sense that it aims at preserving a holistic view on the individual, but it aims at offering tools and ideas to investigate the interconnectedness of interpersonal or social interactions with multilayered domains of activity such as the organic or the sensorimotor. In this way, we do not see the individual as the fundamental or ultimate atomic element that composes the system’s network. Instead, the individual is seen as the attempt of gaining coherence between intertwined processes at multiple scales. We can assume, then, multiple and non-concentric levels of interiority/exteriority composing the individual. Accordingly, the enactive approach advocates for a pluralist perspective on treatment (Fuchs, 2009), acknowledging the efficacy of pharmacological interventions, body-oriented therapies, psychotherapy or socio-community interventions.

As a final remark, Simondon’s philosophy suggests a processual and relational ontology that is in line with the enactive principles and proposes a technical corpus that allows us to develop and enrich enactive perspectives on mental disorders. At the same time, we did not exploit the vast philosophy of Simondon, which offers more concepts (e.g., transduction) that can further complement the enactive framework and might be potentially useful in describing mental disorders. Some of the consequences that we derive from this theoretical dialogue are already present in other approaches. For instance, preventive medicine and allostatic perspectives on psychological processes have already made the claim that not only adaptation counts to health. The concept of adaptivity requires transformation in self-organization and/or transformation of the environment (Kirmayer et al., 2011; Menatti et al., 2022). In the same vein, phenomenological perspectives advocate looking at pathology as existential conditions of life processes that demand changes (Lindsey, 1996; Ratcliffe and Broome, 2012; Carel, 2013). The enactive approach presented here allows us to bring all of these claims and intuitions together into a unified conceptual and theoretical framework. Further research should be aimed at the operationalization of some concepts developed here in a rather loose manner (e.g., *tension*) and their application in the field of cognitive sciences and health research. This work is a preliminary step showing the potential applications of enactive concepts and ideas in mental disorders that hopefully will inspire future research.

## Data availability statement

The original contributions presented in this study are included in the article/supplementary material, further inquiries can be directed to the corresponding author.

## Author contributions

All authors contributed equally to the conceptual analysis, theoretical reflection, writing of the article and manuscript revision, and they read and approved the submitted version.

## Footnotes

1 Indeed, some authors in recent enactive works (Di Paolo et al., 2018; Di Paolo and De Jaegher, 2022) are reluctant to use the term “the body” because it leads to a generalized and uniform idea of the body that disregards individual differences in terms of gender, race, ability, and so on. This is why the plural term “bodies” better reflects their concern to particular, diverse, and concrete bodies.

2 It is worth mentioning that Simondon, despite being Canguilhem and Merleau-Ponty’s student, did not explicitly address the problem of health and pathology. Although he offered some thoughts on anxiety and psychopathology, his main aim was to elaborate a process-oriented metaphysics to understand the emergence of individuals in relation to their associated milieu, that is, the individuation of all sorts of existing entities (physical, organic, psychic and collective). This process perspective on individuation helps us to understand human beings as continuously self-individuating beings that can undergo different kinds of difficulties and breakdowns in this process. In this work, psychopathology will be understood against the background of this open and active process of self-individuation of human beings.

3 This idea is captured by the *life-mind continuity* thesis (Thompson, 2010; Wheeler, 2011; Di Paolo et al., 2017) which proposes that biological and mental phenomena are distinct but deeply interrelated in a complex and non-reductionist manner. It assumes that the same organizational principles that rule life can systematically be extended to account for mental phenomena.

4 For a more exhaustive presentation of Simondon’s philosophy, we address the reader to Scott (2014) and Bardin (2015).

5 Origination of new norms and the emergence of novelty (invention) is hampered by a notion of information limited to the Mathematical Theory of Communication proposed by Shannon (1948). According to this view, information is constrained to uncertainty and it flows within closed systems that include feedback loops while the transmission medium remains unchanged. For Simondon (who defended his thesis *Individuation in Light of Notions of Form and Information* in 1958), information cannot be reduced to the transmission of a signal between two fixed entities. Instead, he proposes the transductive operation, i.e., what is transmitted is a pattern of change that modifies the medium itself—for instance the crystal lattice that extends from the germ along the crystallization axis transforming the solution. This conception of information gives room to invention and historicity in the system (Iliadis, 2013).

6 For instance, think in the opposite role of hormones insulin and glucagon in glycemia. The absence of any of these hormones would be fatal for the organism. Both are necessary to regulate sugar levels in the bloodstream and maintain them in homeostatic ranges. However, these hormones are immersed in a myriad of processes that take in part in glycemia regulation, and looking only at them and their feedback loops is a simplification that might be useful in certain cases but that also has limitations.

7 A clear example is technical normativity, where the production and use of technical objects yields to the emergence of a new normativity in the collective (Bardin, 2015). Artifacts shape collective and individual agents’ norms of actions, habits and action-perception patterns.

8 The tension generated by reflective and pre-reflective forms of experience may be particularly relevant to analyze therapeutic interventions (García, 2021), but a thorough analysis of reflective/pre-reflective processes goes beyond the scope of this article.
